# Russian forest sequesters substantially more carbon than previously reported

**DOI:** 10.1038/s41598-021-92152-9

**Published:** 2021-06-17

**Authors:** Dmitry Schepaschenko, Elena Moltchanova, Stanislav Fedorov, Victor Karminov, Petr Ontikov, Maurizio Santoro, Linda See, Vladimir Kositsyn, Anatoly Shvidenko, Anna Romanovskaya, Vladimir Korotkov, Myroslava Lesiv, Sergey Bartalev, Steffen Fritz, Maria Shchepashchenko, Florian Kraxner

**Affiliations:** 1 Center for Forest Ecology and Productivity of the Russian Academy of Sciences, Profsoyuznaya 84/32/14, Moscow, Russia 117997; 2 International Institute for Applied Systems Analysis, 2361 Laxenburg, Austria; 3 Siberian Federal University, Svobodny pr, Krasnoyarsk, Russia 660041; 4 School of Mathematics and Statistics, University of Canterbury, Christchurch, 8041 New Zealand; 5 FSBI Roslesinforg, Federal Forestry Agency, Volgogradsky pr. 45, Moscow, Russia 109316; 6 Federal Forestry Agency, Pyatnitskaya, 59/19, Moscow, Russia 115184; 7 Russian Institute of Continuous Education in Forestry, Institutskaya 17, Pushkino, Russia 141200; 8 Gamma Remote Sensing, 3073 Gümligen, Switzerland; 9 V.N. Sukachev Institute of Forest, Siberian Branch of the Russian Academy of Science, Academgorodok 50(28), Krasnoyarsk, Russia 660036; 10 Yu. A. Izrael Institute of Global Climate and Ecology, Glebovskaya 20B, Moscow, Russia 107258; 11 Space Research Institute of the Russian Academy of Sciences, Profsoyuznaya 84/32, Moscow, Russia 117997

**Keywords:** Climate-change impacts, Forest ecology, Forestry

## Abstract

Since the collapse of the Soviet Union and transition to a new forest inventory system, Russia has reported almost no change in growing stock (+ 1.8%) and biomass (+ 0.6%). Yet remote sensing products indicate increased vegetation productivity, tree cover and above-ground biomass. Here, we challenge these statistics with a combination of recent National Forest Inventory and remote sensing data to provide an alternative estimate of the growing stock of Russian forests and to assess the relative changes in post-Soviet Russia. Our estimate for the year 2014 is 111 ± 1.3 × 10^9^ m^3^, or 39% higher than the value in the State Forest Register. Using the last Soviet Union report as a reference, Russian forests have accumulated 1163 × 10^6^ m^3^ yr^-1^ of growing stock between 1988–2014, which balances the net forest stock losses in tropical countries. Our estimate of the growing stock of managed forests is 94.2 × 10^9^ m^3^, which corresponds to sequestration of 354 Tg C yr^-1^ in live biomass over 1988–2014, or 47% higher than reported in the National Greenhouse Gases Inventory.

## Main

Russia has been reporting almost no changes in forested area, growing stock volume (GSV) and biomass to the United Nations Framework Convention on Climate Change (UNFCCC)^1^ and the Food and Agriculture Organization of the United Nations (FAO) Forest Resources Assessment (FRA)^2^ since the collapse of the USSR and the decline in the Soviet Forest Inventory and Planning (FIP) system. According to the State Forest Register (SFR)^3^, which is the main repository of forest information, and national reporting to the FAO FRA^2^, the GSV and the above ground biomass (AGB) increased by 1.1% and 0.6% (Table S1), respectively, during 1990–2015, yet studies using remote sensing (RS) indicate increased vegetation productivity^4^, tree cover (annual rate + 0.417% over 1982–2016)^5^, increased AGB (+ 329 Tg C yr^−1^ over 2000–2007^6^), total biomass (annual rate + 0.44% or + 153 Tg C yr^−1^ over 1990–2007^7^), and forest ecosystem carbon pools (ca + 470 Tg C yr^−1^ over 2001–2019^8^). This inconsistency in estimates can be explained by an information gap that appeared when Russia decided to move from the FIP to another system for the collection of forest information at the national scale – the National Forest Inventory (NFI).

The FIP involves revisiting every forest stand (on the ground for managed forests or using RS techniques for remote non-commercial forests) on a 10–15-year interval, with the measurement of forest parameters combined with the formulation of forest management directives. After the collapse of the USSR, the inventory within the FIP system slowed down substantially. For example, more than 50% of the forest area was surveyed by the FIP more than 25 years ago^9^. For these reasons, the reliability of information on forests in Russia has deteriorated since 1988, which is the year when FIP-based reporting^10^ involved the largest inventory efforts in recent decades. According to this report^10^, the total GSV of Russian forests was 81.7 × 10^9^ m^3^ (without shrubland, bias corrected^11^). This value is used here as a reference to quantify biomass stock changes in Russia with respect to the current decade.

In contrast, NFI is a state-of-the-art inventory system based on a statistical sampling method. It was initiated in 2007 and the first cycle was completed in 2020. The NFI data processing is ongoing, but the first official press release^12^ suggests that Russian forest accumulated 102 × 10^9^ m^3^ over its lifespan until 2014. Once finalized, the NFI will be verified before adoption as the official source of information to the SFR and national reporting. The NFI has received some criticism^13^ because of the relatively sparse sampling employed and the stratification method used, which is partially based on outdated FIP data.

In Russia, the long intervals between consecutive surveys and the difficulty in accessing very remote regions in a timely manner by an inventory system make satellite RS an essential tool for capturing forest dynamics and providing a comprehensive, wall-to-wall perspective on biomass distribution. However, observations from current RS sensors are not suited for producing accurate biomass estimates unless the estimation method is calibrated with a dense network of measurements from ground surveys^14^. Here we calibrated models relating two global RS biomass data products (GlobBiomass GSV^15^ and CCI Biomass GSV^16^) and additional RS data layers (forest cover mask^9^, the Copernicus Global Land Cover CGLS‐LC100 product^17^) with ca 10,000 ground plots (see Material and Methods) to reduce nuances in the individual input maps due to imperfections in the RS data and approximations in the retrieval procedure^18,19^. The combination of these two sources of information, i.e., ground measurements and RS, utilizes the advantages of both sources in terms of: (i) highly accurate ground measurements and (ii) the spatially comprehensive coverage of RS products and methods. The amount of ground plots currently available may be insufficient for providing an accurate estimate of GSV for the country when used alone, but they are the key to obtaining unbiased estimates when used to calibrate RS datasets^20^. The map merging procedure was preferred over a plot-aided direct estimation of GSV or AGB from the RS data because of the usually poor association between biomass measured at inventory plots and remote sensing observables^21^. In addition, models relating biomass and remote sensing observables that are trained with spatially inhomogeneous datasets (Figure S1) tend to be biased in regions not represented by the dataset of the reference biomass measurements.

**Figure 1:**
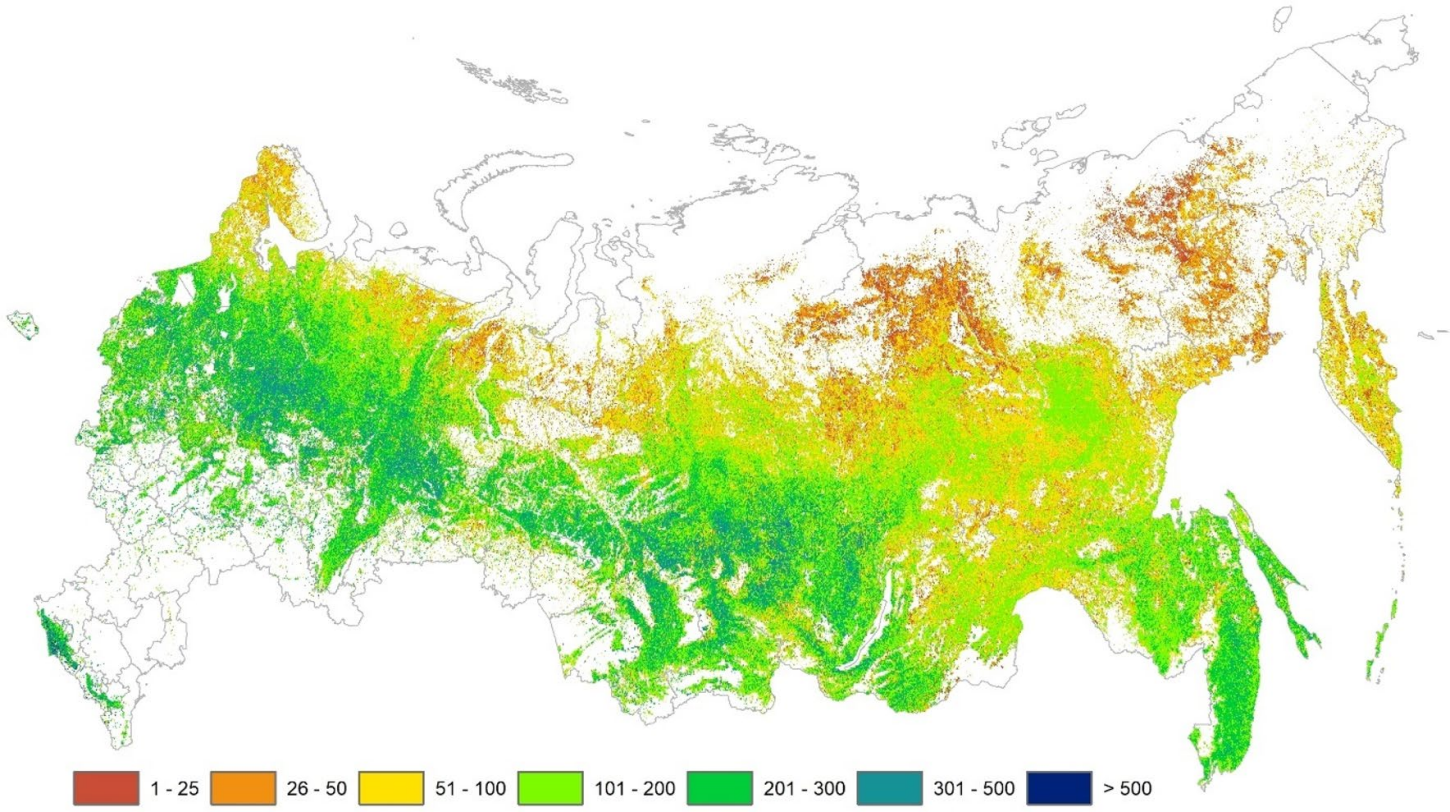
Predicted mean forest growing stock volume (m^3^ ha^-1^) for the year ca 2014 (Generated by Esri ArcGIS Desktop v.10.7, URL: https://desktop.arcgis.com/en/arcmap/).

We estimate the total GSV of Russia for the year 2014 for the official forested area (713.1 × 10^6^ ha) to be 111 ± 1.3 × 10^9^ m^3^, which is 39% higher than the 79.9 × 10^9^ m^3^ (excluding shrubland) figure reported in the SFR^3^ for the same year. An additional 7.1 × 10^9^ m^3^ or 9% were found due to the larger forested area (+ 45.7 10^6^ ha) recognized by RS^9^, following the expansion of forests to the north^22^, to higher elevations, in abandoned arable land^23^, as well as the inclusion of parks, gardens and other trees outside of forest, which were not counted as forest in the SFR. Based on cross-validation, our estimate at the regional level (81 regions of Russia – Table S2, Figure S2) is unbiased. The standard error varied from 0.6 to 17.6% depending on the region. The median error was 1.6%, while the area weighted error was 1.2%. The predicted GSV (Fig. 1) with associated uncertainties is available here (https://doi.org/10.5281/zenodo.3981198) as a GeoTiff at a spatial resolution of 3.2 arc sec. (ca 0.5 ha).

Houghton et al.^24^ estimated forest biomass based on RS and FIP data in Russia for the year 2000. Average forest biomass density varied between 80.6 and 88.2 Mg ha^-1^ depending on which forest mask was used. Our estimate for the year 2014 of 107 Mg ha^-1^ (using the conversion factor of GSV to AGB from^24^ 0.6859) is 21–33% higher than the one by Houghton et al., but this is consistent with expected biomass increases over time, i.e., 14 years after the Houghton et al. estimate.

Assuming an unchanged total forest area (721.7 × 10^6^ ha) in 1988 and 2014, we conclude that Russian forests have accumulated 1,163 × 10^6^ m^3^ yr^-1^ or 407 Tg C yr^-1^ in live biomass of trees on average over 26 years. This gives an average GSV change rate of + 1.61 m^3^ ha^-1^ yr^-1^ or + 0.56 t C ha^-1^ yr^-1^. The sequestration rate obtained, however, should be treated with caution because different methods have been applied in 1988 and 2014 (see “Caveats and Limitations” section). To provide some context for the magnitude of these numbers, one can compare the Russian forest gain to the net GSV losses in tropical forests over the period 1990–2015 according to FAO FRA^25^ (-1,033 × 10^6^ m^3^ yr^-1^ in the regions with a negative trend: South and Central America, South and Southeast Asia, and Africa). A similar divergence in the carbon sink between Tropical and Boreal forest was recognized by Tagesson et al.^26^.

In terms of carbon stock change, our estimates are substantially higher than those reported by Pan et al.^7^ for 1990–2007 (+ 153 Tg C yr^-1^) based on FIP data. The biomass carbon estimates by Liu et al.^6^ are instead in line with our results. There is an increase in the annual rate of AGB in Russia of + 329 Tg C yr^−1^ (annual variation from 214 to 400 Tg C yr^−1^) over 2000–2007^6^. Interestingly, another boreal country – Canada – has demonstrated neutral or negative trends (from 0 to -14 Tg C yr^−1^) for the same time span using the same estimation method^6^.

**Figure 2:**
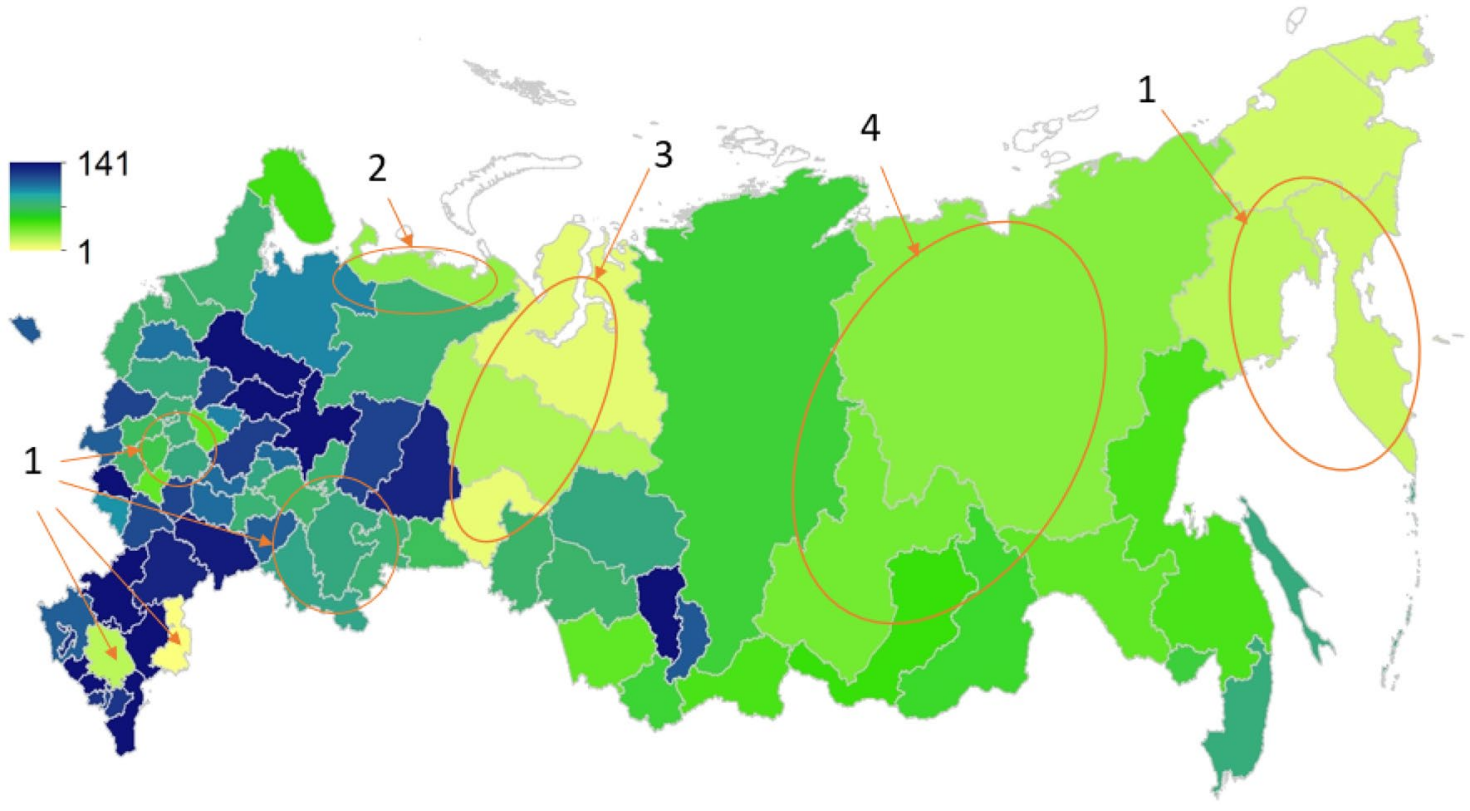
Change in growing stock volume (m^3^ ha^-1^) from 1988 to 2014 (average over administrative regions) (Generated by Esri ArcGIS Desktop v.10.7, URL: https://desktop.arcgis.com/en/arcmap/). These changes can be categorized into: 1—significant increase in air temperature and drought; 2—substantially increased forest area, which lowers the average GSV density; 3—least (not significant) temperature increase; 4—increase of disturbances: wildfire and harvest (southern part), which offsets the climate stimulation effect.

We can observe different spatial patterns in the change in the GSV density between 1988 (FIP^10^, bias corrected^11^) and 2014 (our estimate), which can be explained by climate change, CO_2_ fertilisation and changes in disturbance regimes (Fig. 2). The average linear trend in the annual temperature increase during 1976–2014 in Russia is + 0.45 °C per 10 years^27^. The temperature increase is statistically significant in every region except for western Siberia (Fig. 2–3). Significantly increased temperature extremes and an increase in the number of days without precipitation is observed in the south of European Russia, Baikal, Kamchatka, and Chukotka^27^ (Fig. 2–1). Some regions in the south of the European part of Russia are colored in dark blue, but they, as a rule, have a small share of forested area, which is often linked to water bodies and, therefore, suffers less from increased drought (Fig. 2–1). Central and eastern Siberia suffer from an increase in disturbances, which offsets the climate stimulation effect (Fig. 2–4). The forested area in the Nenets region (Fig. 2–2) is 4 times larger in 2014 based on the RS forest mask compared to the SFR in 1988 (where forest was accounted for up until a certain latitude at that time), where the increase in area resulted in a decrease in the average GSV.

Focusing specifically on national reporting of managed forest to the UNFCCC, 72% of forested area in Russia is considered to be managed^1^. We multiplied the GSV density by the managed forest area for each administrative region (Table S3). The difference in GSV estimation (between ours and the one from the SFR report) is 23.6 × 10^9^ m^3^ (Table S3) or 33% higher. From the GSV of managed forests in 2014 and based on the same area in 1988, we can estimate the sequestration rate of live biomass of managed forests as 354 Tg C yr^-1^ , which is considerably higher than the figure of 230 Tg C yr^-1^ in the current report^1^.

This proof of concept demonstrates the relevance of complementing recent NFI data with remote sensing map products. Our study demonstrates that the already considerable value of forest inventory data can be further enhanced in a forest resources mapping scenario. In addition, we seek to promote greater access to these data by opening up their access to the larger scientific community. Through the integration of RS estimates of GSV and forest inventory data from Russia, we confirm that carbon stocks increased substantially during the last few decades in contrast to the figures provided in official national reporting. Russian forests play an even more important global role in carbon sequestration than previously thought, where the increase in growing stock is of the same magnitude as the net losses in tropical forests over the same time period.

## Material and methods

### Ground data

Measurements of GSV consisted of observations from forest plots from both the NFI and the Forest Observation System (FOS)^28^, which were used to ground truth the model by relating inventory measurements and RS data products. The NFI implements a random stratified sampling of forests. The plots have a circular shape and cover an area of 0.05 ha^13^. A full set of inventory plots from 10 regions in Russia (Table S2) was available for the first time to undertake research studies outside of the NFI. The FOS^28^ offers free access to research forest plots with a size of 0.25 ha or larger. In total, 8,988 NFI (after data screening and verification, see section “Forest plot data screening”) and 100 FOS plots were gathered (Figure S1). The dataset covers the full range of GSV (Figure S2), all climatic zones and a major diversity of forest types. The calibrating dataset is described in Table S4 and available in csv format in the Supplementary Information. The ground measurements were collected between 2008 and 2019 (with the median falling in 2014).

As in many other countries, the NFI data (with plot coordinates) are restricted for sharing and use. For the first time, we obtained access to a portion of the primary NFI data with precise location information under the condition that the initial data processing was physically undertaken at the location of the authorized division (“Roslesinforg”) of the Federal Forestry Agency.

### Remote sensing data products and other maps

We used several RS-based maps to predict the spatial distribution of GSV in Russia for around the epoch 2014 as follows:

- The global GlobBiomass map of GSV^15,19^ is based on the Phased Array-type L-band Synthetic Aperture Radar (PALSAR) onboard the Advanced Land Observing Satellite (ALOS) satellite, and the Advanced Synthetic Aperture Radar (ASAR) onboard the Environmental Satellite (Envisat) observations acquired around the year 2010 with a spatial resolution of the final product of 3.2 arc sec. (0.496 ha for Russia on average), units m^3^ ha^-1^. The map is obtained from a physically-based model that relates GSV to the input remote sensing observations. To estimate the parameters of the model, a so-called self-calibration approach based on image statistics was applied^19^, thus overcoming the use of reference GSV measurements from field inventory or existing maps.
- The global Climate Change Initiative (CCI) Biomass map of GSV^16^ is based on ALOS-2 PALSAR-2 (2015–2017) and the Sentinel-1 data, acquired in 2017. It has the same units, spatial resolution and generating algorithm as the GlobBiomass map.
- The Copernicus Global Land Cover CGLS‐LC100 product^17^ is based on optical data acquired around the year 2015 and has a similar resolution (3.6 arc sec.). The dataset stratifies forests into four classes: evergreen needleleaf, deciduous needleleaf, deciduous broadleaf and mixed forest.
- The forest mask for the year 2015: is a hybrid product based on the methodology described in^9^. It has a 3.2 arc sec. spatial resolution.
- The ecological zone map ^29^ includes classes of forest-tundra, north taiga, middle taiga, south taiga, temperate forest, and forest-steppe.

In addition, results were evaluated using a map of 81 administrative regions (Table S2, Table S3).

### Forest plot data screening

To calibrate the RS maps with the aid of the inventory measurements, it was necessary to ensure that the plot measurements and the map values were consistent. Very high-resolution imagery provided by Google Earth was used to filter out records that were characterized by obvious contradictions in terms of biomass values and forest cover. Figure S3a shows an example of a forest felled in 2009. The sample plot was measured in 2008 before the disturbance while the RS data were collected in 2010 after the disturbance. The sample plot in Figure S3b is situated at the edge of the forest and is not representative of the RS pixel, which covers partly non-forested area. As a result of this data screening process, up to 10% of plots in some regions were discarded. The plot-to-pixel comparison of GSV values (Figure S4) still reveals some substantial divergences, which can be attributed to the following reasons:

1. The size of the NFI plot is about 10% of the area of a GlobBiomass pixel (i.e., 0.05 ha vs. ca 0.5 ha).
2. The estimations made on the ground and remotely were not simultaneous.
3. The method used to estimate GSV based on RS data implements a regional cut-off level to avoid unrealistic estimates and biases^19^. These cut-off levels imply that extreme GSV values are strongly underestimated.

### Growing stock prediction model

We used 20-fold cross-validation to compare the predictive fit of several models to calibrate the RS maps with ground measurements. Based on the model performance statistics such as mean error (ME), mean absolute error (MAE), and mean squared error (MSE) (see Table S5), the following linear model was selected:

$$E\left( {GSV_{{GT}} } \right) = \left( {a_{{0,zone}} + {\text{ }}b_{{0,PFT}} } \right) + \left( {a_{{1,zone}} + {\text{ }}b_{{1,PFT}} } \right) \times GSV_{{GB}} + \left( {a_{{2,zone}} + {\text{ }}b_{{2,PFT}} } \right) \times GSV_{{CCI}} + \left( {a_{{3,zone}} + {\text{ }}b_{{3,zone}} } \right){\text{ }} \times {\text{ }}GSV_{{GB}} \times {\text{ }}GSV_{{CCI}} ,$$

where GSV_GT_ – GSV estimates on the ground sample plots, m^3^ ha^-1^; GSV_GB_ – GlobBiomass GSV, m^3^ ha^-1^; GSV_CCI_ – CCI Biomass GSV, m^3^ ha^-1^; zone – bioecological zone (forest-tundra, north taiga, middle taiga, south taiga, temperate forest, forest-steppe); PFT – forest type (evergreen needleleaf, deciduous needleleaf, deciduous broadleaf and mixed forest).

Since the linear model allows for negative predictions, these negative values were set to zero. However, it should be noted, that only 0.5% of points in the calibrating dataset (ground plots) and only 1.7% of the pixels in the testing dataset (entire country) produced negative predictions, implying negligible bias.

Recognizing that the frequency distribution of the GSV and AGB measurements varied from region to region and that they might have differed from the respective frequency distributions in the calibrating datasets, we also fitted a weighted linear regression model. The weighted linear regression fits parameters such that the weighted sum of errors is zero. It can thus be used to ensure that the estimate for the average (or the sum) of predictions over a certain area is unbiased. The weights were based on the relative frequencies of GSV in the calibrating data and the administrative region, one at a time, evaluated in bins of width 10 from 0 to 1000.

Because the residuals of the resulting model displayed strong heteroscedasticity, the estimated standard errors for the regression parameters could not be used to produce confidence intervals for the predictions. We have, therefore, used 1000 bootstrapped estimates to obtain the overall estimates, standard errors and 95% confidence intervals for the administrative area-specific GSV density per ha (see Supplementary S2. R-script fitting the model and cross-validation).

### Growing stock to biomass conversion factor

We use biomass conversion and expansion factors from Schepaschenko et al.^30^ for the entire country in order to compare with other independent studies in the situation where they do not provide GSV estimates. These factors consider species, age, stocking and the forest productivity distribution of Russian forests^30^. The conversion factors are as follows:

- GSV to total live biomass carbon of trees: 0.35035
- GSV to AGB carbon: 0.27923
- GSV to AGB: 0.56131
- Root-to-shoot ratio: 0.288
- We assumed that carbon content in woody biomass is around 50% and 45% for the foliage.

## Caveats and limitations

This analysis employed the largest amount of forest sample plots among any other remote sensing assessments for Russia. However, every plot represents quite large forest areas (country forest area divided by number of ground plots = 78 × 10^3^ ha) at the country scale and there are some large regions in Northern Asia that are not covered (Figure S1). Currently, only a portion of the NFI data (ca 11%) were made available exclusively for this proof of concept. However, the sample plots used cover the full range of biomass values (Figure S2), and they represent all bioclimatic zones and the majority of forest types. More calibrating data might improve the spatial accuracy, but they were not available at the time when this manuscript was prepared. By demonstrating the value of the sample plot data with RS, we hope to facilitate the further opening up of these datasets in the future for the wider scientific community.

The National Forest Inventory is currently finalizing its first cycle, so all the plots have been measured only once. Subsequent long-term observations on these permanent plots would help to quantify changes in biomass and other carbon pools more accurately.

The estimates of GSV in 1988 and in 2014 used different methods, which might introduce an unknown bias. For this reason, the estimates of GSV dynamics and carbon sequestration rates need to be treated with caution. However, the 1988 USSR forest assessment is the most reliable reference point. The massive FIP program started in the Soviet Union in the late 1940s with the first complete country report produced in 1961, followed by national reports every 5 years based on repeated observations. The quality of the FIP substantially improved over time. Shvidenko and Nilsson^11^ analyzed the FIP method and reports based on numerous independent regional validation exercises and introduced a regional bias correction. They have shown that the 1988 report minimized the bias of the country average GSV over the entire previous period. Both the 1988 and 2014 estimates are based on the best available knowledge and rely on the vast field and RS measurements made.

Our GSV estimates for the year 2014 might include a portion of standing dry wood (snags), which is not possible to quantify. We excluded snags on sample plots. However, the ratio of snag volume to GSV on the NFI sample plots was 12% while an independent study by Shvidenko et al.^31^ estimated the weighted average ratio for Russian forests at 16%. Another research study ^32^ in Central Siberia reports the ratio of snag volume to GSV at 4–11% in middle taiga up to 17–19% in northern taiga. In general, snags are less recognizable using remote instruments because of reduced crown elements. However, a portion of snags might lead to slight overestimation of GSV by our method.

## Supplementary Information

Supplementary file 1.

Supplementary file 2.

## Data Availability

The data used for this study are either publicly available (see Material and Methods section) or can be found in the Supplementary Information.

## Code availability

The R script used in this study is given in the Supplementary Information.
