# Supplementary material for: Russian forest sequesters substantially more carbon than previously reported
Source: Sci Rep. 2021 Jun 17;11:12825. doi: 10.1038/s41598-021-92152-9 (PMC8211780; doi:10.1038/s41598-021-92152-9)
Supplement: Supplementary file 2 [file 41598_2021_92152_MOESM2_ESM.docx]

Supplementary

Russian forest sequesters substantially more carbon than previously reported

Dmitry Schepaschenko et al.

Table S1. FAO FRA 2015 ^1^

| Indicator | **1990** | **2000** | **2005** | **2010** | **2015** | **Change  1990-2015, %** |
| --- | --- | --- | --- | --- | --- | --- |
| Stocked forest land (10^3^ ha) | 808949.9 | 809268.5 | 808790 | 815135.6 | 814930.5 | +0.7 |
| Growing stock (10^6^ m^3^) | 80039.64 | 80270.39 | 80479.05 | 81522.85 | 81488.06 | +1.8 |
| Above ground biomass (10^9^ t) | 52103.2 | 51471.1 | 51573.5 | 52000 | 52400 | +0.6 |

Table S2. Results of GSV estimation by administrative regions of Russia

| ID | Administrative region | # ground  plots | Forested area,  10^3^ ha | GSV,  m³ ha^-1^ | Std. dev.,  m³ ha^-1^ | 95% min,  m³ ha^-1^ | 95% max,  m³ ha^-1^ | GSV, 10^6^ m³ | GSV change, m³ ha^-1^ yr^-1^ |
| --- | --- | --- | --- | --- | --- | --- | --- | --- | --- |
| 14 | Belgorod oblast |  | 307 | 201 | 5.6 | 191 | 213 | 62 | 2.7 |
| 15 | Bryansk oblast | 9 | 1'443 | 233 | 2.52 | 228 | 238 | 336 | 3.2 |
| 17 | Vladimir oblast |  | 1'703 | 179 | 2.51 | 174 | 184 | 305 | 1.1 |
| 20 | Voronezh oblast |  | 505 | 222 | 7.55 | 208 | 238 | 112 | 3.4 |
| 24 | Ivanovo oblast |  | 1'068 | 235 | 2.29 | 231 | 240 | 251 | 2.9 |
| 28 | Tver oblast |  | 5'245 | 224 | 2.34 | 220 | 229 | 1'175 | 2.3 |
| 29 | Kaluga oblast |  | 1'492 | 218 | 2.09 | 214 | 222 | 325 | 2.0 |
| 34 | Kostroma oblast |  | 4'704 | 278 | 2.63 | 273 | 283 | 1'308 | 4.8 |
| 38 | Kursk oblast |  | 277 | 227 | 8.28 | 212 | 243 | 63 | 5.3 |
| 42 | Lipetsk oblast |  | 250 | 189 | 5.19 | 178 | 198 | 47 | 1.1 |
| 46 | Moscow oblast | 20 | 2'280 | 241 | 3.78 | 234 | 249 | 549 | 2.2 |
| 54 | Oryol oblast |  | 289 | 182 | 4.14 | 175 | 191 | 53 | 2.1 |
| 61 | Ryazan oblast |  | 1'221 | 201 | 2.4 | 196 | 206 | 245 | 2.3 |
| 66 | Smolensk oblast | 1 | 2'430 | 218 | 2.31 | 213 | 222 | 530 | 3.5 |
| 68 | Tambov oblast |  | 408 | 229 | 5.33 | 219 | 240 | 93 | 3.5 |
| 70 | Tula oblast |  | 442 | 179 | 3.77 | 172 | 187 | 79 | 1.8 |
| 78 | Yaroslavl oblast | 714 | 1'911 | 228 | 2.17 | 224 | 233 | 436 | 3.5 |
| 11 | Arkhangelsk oblast | 2 | 22'422 | 184 | 1.62 | 181 | 187 | 4'126 | 2.8 |
| 19 | Vologda oblast |  | 10'967 | 256 | 1.6 | 253 | 260 | 2'808 | 4.4 |
| 23 | Nenets a.okr. |  | 915 | 107 | 3.05 | 101 | 113 | 98 | 0.8 |
| 27 | Kaliningrad oblast | 1 | 345 | 240 | 2.59 | 236 | 245 | 83 | 3.3 |
| 41 | Leningrad oblast |  | 5'206 | 226 | 2.03 | 222 | 230 | 1'177 | 2.1 |
| 47 | Murmansk oblast | 280 | 5'316 | 74 | 1.43 | 71 | 77 | 393 | 1.3 |
| 49 | Novgorod oblast |  | 3'902 | 235 | 2.33 | 230 | 239 | 917 | 3.0 |
| 58 | Pskov oblast |  | 3'309 | 201 | 2.57 | 196 | 206 | 665 | 2.1 |
| 86 | Karelia Republic |  | 9'606 | 147 | 1.26 | 144 | 149 | 1'412 | 2.1 |
| 87 | Komi Republic | 1653 | 30'905 | 153 | 1.27 | 151 | 156 | 4'728 | 2.2 |
| 3 | Krasnodar Krai | 13 | 1'678 | 285 | 24.45 | 242 | 341 | 478 | 3.2 |
| 12 | Astrakhan oblast |  | 75 | 74 | 13 | 51 | 103 | 6 | 0.0 |
| 18 | Volgograd oblast |  | 406 | 168 | 9.94 | 150 | 189 | 68 | 3.8 |
| 60 | Rostov oblast |  | 245 | 180 | 10.91 | 159 | 203 | 44 | 5.1 |
| 79 | Adigea Republic | 194 | 323 | 299 | 26 | 252 | 353 | 97 | 3.7 |
| 85 | Kalmykia Republic |  | 2 | 147 | 9.94 | 130 | 168 | 0 | 4.8 |
| 7 | Stavropol Krai |  | 108 | 218 | 10.28 | 199 | 239 | 23 | 0.5 |
| 9 | Ingushetia Republic |  | 111 | 255 | 11.59 | 234 | 279 | 28 | 3.5 |
| 13 | Chechnya Republic |  | 360 | 252 | 6.46 | 239 | 264 | 91 | 3.6 |
| 82 | Dagestan Republic |  | 397 | 227 | 9.81 | 209 | 247 | 90 | 4.3 |
| 83 | Kabardino-Balkaria Republic |  | 216 | 265 | 8.85 | 248 | 283 | 57 | 4.2 |
| 90 | North-Ossetia-Alania Republic |  | 224 | 275 | 8.87 | 258 | 293 | 62 | 3.7 |
| 91 | Karachay-Cherkessia Republic |  | 479 | 337 | 21.15 | 296 | 379 | 161 | 5.4 |
| 22 | Nizhny Novgorod oblast |  | 4'184 | 231 | 2.04 | 227 | 235 | 966 | 3.5 |
| 33 | Kirov oblast |  | 8'205 | 251 | 1.81 | 247 | 254 | 2'059 | 4.3 |
| 36 | Samara oblast |  | 704 | 208 | 8.59 | 192 | 225 | 146 | 3.2 |
| 53 | Orenburg oblast |  | 519 | 185 | 9.09 | 168 | 204 | 96 | 2.4 |
| 56 | Penza oblast |  | 1'132 | 219 | 5.39 | 209 | 230 | 248 | 3.1 |
| 57 | Perm oblast |  | 12'504 | 230 | 1.5 | 227 | 233 | 2'876 | 3.5 |
| 63 | Saratov oblast |  | 610 | 200 | 12.68 | 177 | 226 | 122 | 4.0 |
| 73 | Ulyanovsk oblast |  | 1'151 | 210 | 4.34 | 202 | 218 | 242 | 2.1 |
| 80 | Bashkortostan Republic |  | 6'079 | 199 | 2.63 | 194 | 204 | 1'210 | 2.3 |
| 88 | Mari-El Republic |  | 1'417 | 219 | 2.29 | 214 | 223 | 310 | 3.1 |
| 89 | Mordovia Republic |  | 847 | 213 | 4.33 | 205 | 223 | 181 | 3.1 |
| 92 | Tatarstan Republic |  | 1'280 | 192 | 4.06 | 184 | 200 | 246 | 2.1 |
| 94 | Udmurt Republic |  | 2'192 | 209 | 2.19 | 205 | 214 | 458 | 2.1 |
| 97 | Chuvash Republic |  | 647 | 203 | 2.76 | 198 | 209 | 131 | 2.3 |
| 37 | Kurgan oblast |  | 1'407 | 174 | 4.83 | 165 | 184 | 245 | 1.9 |
| 65 | Sverdlovsk oblast | 1914 | 14'783 | 243 | 1.38 | 241 | 246 | 3'592 | 3.8 |
| 71 | Tyumen oblast |  | 7'807 | 164 | 2.48 | 159 | 169 | 1'280 | 0.2 |
| 72 | Khanty-Mansi a.okr. |  | 27'409 | 136 | 1.25 | 134 | 139 | 3'728 | 0.6 |
| 74 | Yamalo-Nenets a.okr. | 659 | 15'404 | 71 | 1.32 | 68 | 73 | 1'094 | 0.2 |
| 75 | Chelyabinsk oblast |  | 2'675 | 200 | 2.47 | 195 | 205 | 535 | 2.2 |
| 1 | Altai Krai |  | 3'846 | 189 | 2.65 | 184 | 195 | 727 | 1.1 |
| 2 | Altai Republic |  | 4'058 | 204 | 5.09 | 195 | 215 | 828 | 1.6 |
| 4 | Krasnoyarsk Krai | 46 | 99'902 | 170 | 1.29 | 167 | 173 | 16'983 | 1.6 |
| 25 | Irkutsk oblast | 3 | 59'805 | 193 | 1.71 | 190 | 197 | 11'542 | 1.0 |
| 32 | Kemerovo oblast |  | 6'531 | 248 | 2.83 | 243 | 253 | 1'620 | 5.3 |
| 50 | Novosibirsk oblast |  | 4'955 | 164 | 3.74 | 157 | 172 | 813 | 2.1 |
| 52 | Omsk oblast | 456 | 4'659 | 179 | 2.35 | 175 | 184 | 834 | 2.1 |
| 69 | Tomsk oblast |  | 19'002 | 218 | 1.88 | 214 | 221 | 4'142 | 2.3 |
| 93 | Tuva Republic |  | 7'559 | 184 | 3.27 | 178 | 191 | 1'391 | 1.3 |
| 95 | Khakassia Republic |  | 3'297 | 211 | 2.93 | 206 | 217 | 696 | 3.3 |
| 5 | Primorski Krai | 4 | 13'127 | 218 | 2.32 | 214 | 223 | 2'862 | 2.2 |
| 8 | Khabarovsk Krai | 1967 | 51'445 | 146 | 1.36 | 143 | 149 | 7'511 | 1.3 |
| 10 | Amur oblast |  | 21'778 | 125 | 1.99 | 121 | 129 | 2'722 | 1.1 |
| 21 | Jewish a. oblast |  | 1'664 | 160 | 2.87 | 154 | 165 | 266 | 1.7 |
| 30 | Kamchatka oblast | 577 | 16'482 | 83 | 2.37 | 79 | 88 | 1'368 | 0.4 |
| 35 | Chukotka a.okr. |  | 3'069 | 41 | 1.81 | 37 | 44 | 126 | 0.3 |
| 44 | Magadan oblast |  | 14'629 | 42 | 2.42 | 38 | 47 | 614 | 0.5 |
| 64 | Sakhalin oblast |  | 6'117 | 191 | 1.3 | 189 | 194 | 1'168 | 2.3 |
| 76 | Zabaykalsky Krai |  | 26'131 | 138 | 2.11 | 133 | 142 | 3'606 | 1.5 |
| 81 | Buryatia Republic | 2 | 19'015 | 151 | 1.8 | 148 | 155 | 2'871 | 1.4 |
| 98 | Sakha Republic | 580 | 137'276 | 89 | 1.8 | 85 | 92 | 12'218 | 0.9 |
|  | Entire Russia | 9088 | 758'793 | 156 | 1.93 | 152 | 160 | 118'255 | 1.6 |

Table S3. Regional comparison of our and SFR 2015 managed forest estimation

| ID | Admin region | Area of managed  forest, 10^3^ ha | Managed SFR 2015 | | Our estimation | | Difference, 10^6^ m^3^ |
| --- | --- | --- | --- | --- | --- | --- | --- |
|  |  |  | GSV, m^3^ ha^-1^ | GSV, 10^6^ m^3^ | GSV, m^3^ ha^-1^ | GSV, 10^6^ m^3^ |  |
| 14 | Belgorod oblast | 226 | 195 | 44 | 201 | 46 | 1 |
| 15 | Bryansk oblast | 1'144 | 196 | 224 | 233 | 267 | 42 |
| 17 | Vladimir oblast | 1'483 | 181 | 268 | 179 | 266 | -3 |
| 20 | Voronezh oblast | 369 | 178 | 66 | 222 | 82 | 16 |
| 24 | Ivanovo oblast | 984 | 173 | 171 | 235 | 232 | 61 |
| 28 | Tver oblast | 4'522 | 166 | 752 | 224 | 1'014 | 262 |
| 29 | Kaluga oblast | 1'301 | 200 | 260 | 218 | 284 | 24 |
| 34 | Kostroma oblast | 4'473 | 162 | 723 | 278 | 1'243 | 520 |
| 38 | Kursk oblast | 232 | 186 | 43 | 227 | 53 | 9 |
| 42 | Lipetsk oblast | 175 | 177 | 31 | 189 | 33 | 2 |
| 46 | Moscow oblast | 1'914 | 212 | 406 | 241 | 461 | 55 |
| 54 | Oryol oblast | 125 | 196 | 25 | 182 | 23 | -2 |
| 61 | Ryazan oblast | 836 | 184 | 154 | 201 | 168 | 14 |
| 66 | Smolensk oblast | 2'041 | 166 | 338 | 218 | 444 | 106 |
| 68 | Tambov oblast | 360 | 195 | 70 | 229 | 83 | 12 |
| 70 | Tula oblast | 276 | 243 | 67 | 179 | 50 | -18 |
| 78 | Yaroslavl oblast | 1'410 | 173 | 244 | 228 | 322 | 79 |
| 11 | Arkhangelsk oblast | 22'398 | 119 | 2'662 | 184 | 4'129 | 1'467 |
| 19 | Vologda oblast | 9'988 | 165 | 1'651 | 256 | 2'561 | 910 |
| 23 | Nenets a.okr. | 191 | 96 | 18 | 107 | 20 | 2 |
| 27 | Kaliningrad oblast | 266 | 195 | 52 | 240 | 64 | 12 |
| 41 | Leningrad oblast | 4'810 | 170 | 819 | 226 | 1'086 | 266 |
| 47 | Murmansk oblast | 5'417 | 44 | 238 | 74 | 400 | 162 |
| 49 | Novgorod oblast | 3'461 | 166 | 573 | 235 | 813 | 240 |
| 58 | Pskov oblast | 2'144 | 157 | 337 | 201 | 432 | 94 |
| 86 | Karelia Republic | 9'546 | 105 | 1'004 | 147 | 1'401 | 398 |
| 87 | Komi Republic | 30'048 | 100 | 3'015 | 153 | 4'608 | 1'593 |
| 3 | Krasnodar Krai | 1'510 | 232 | 350 | 285 | 430 | 81 |
| 12 | Astrakhan oblast | 77 | 87 | 7 | 74 | 6 | -1 |
| 18 | Volgograd oblast | 446 | 72 | 32 | 168 | 75 | 43 |
| 60 | Rostov oblast | 213 | 88 | 19 | 180 | 38 | 20 |
| 79 | Adigea Republic | 287 | 292 | 84 | 299 | 86 | 2 |
| 85 | Kalmykia Republic | 9 | 47 | 0 | 147 | 1 | 1 |
| 7 | Stavropol Krai | 99 | 113 | 11 | 218 | 22 | 10 |
| 9 | Ingushetia Republic | 75 | 162 | 12 | 255 | 19 | 7 |
| 13 | Chechnya Republic | 273 | 168 | 46 | 252 | 69 | 23 |
| 82 | Dagestan Republic | 353 | 123 | 43 | 227 | 80 | 37 |
| 83 | Kabardino-Balkaria Republic | 190 | 185 | 35 | 265 | 50 | 15 |
| 90 | North-Ossetia-Alania Republic | 185 | 212 | 39 | 275 | 51 | 12 |
| 91 | Karachay-Cherkessia Republic | 404 | 218 | 88 | 337 | 136 | 48 |
| 22 | Nizhny Novgorod oblast | 3'538 | 161 | 570 | 231 | 816 | 245 |
| 33 | Kirov oblast | 7'587 | 154 | 1'169 | 251 | 1'901 | 732 |
| 36 | Samara oblast | 659 | 159 | 104 | 208 | 137 | 32 |
| 53 | Orenburg oblast | 472 | 142 | 67 | 185 | 87 | 20 |
| 56 | Penza oblast | 888 | 169 | 150 | 219 | 194 | 45 |
| 57 | Perm oblast | 11'442 | 142 | 1'624 | 230 | 2'634 | 1'010 |
| 63 | Saratov oblast | 619 | 107 | 66 | 200 | 124 | 57 |
| 73 | Ulyanovsk oblast | 908 | 185 | 168 | 210 | 190 | 23 |
| 80 | Bashkortostan Republic | 5'540 | 146 | 810 | 199 | 1'101 | 291 |
| 88 | Mari-El Republic | 1'202 | 167 | 201 | 219 | 263 | 61 |
| 89 | Mordovia Republic | 695 | 179 | 124 | 213 | 148 | 24 |
| 92 | Tatarstan Republic | 1'181 | 167 | 198 | 192 | 226 | 29 |
| 94 | Udmurt Republic | 1'954 | 168 | 328 | 209 | 409 | 81 |
| 97 | Chuvash Republic | 584 | 128 | 75 | 203 | 119 | 44 |
| 37 | Kurgan oblast | 1'543 | 136 | 209 | 174 | 268 | 59 |
| 65 | Sverdlovsk oblast | 13'184 | 159 | 2'094 | 243 | 3'209 | 1'115 |
| 71 | Tyumen oblast | 6'887 | 136 | 938 | 164 | 1'126 | 189 |
| 72 | Khanty-Mansi a.okr. | 28'696 | 114 | 3'286 | 136 | 3'909 | 624 |
| 74 | Yamalo-Nenets a.okr. | 15'344 | 75 | 1'154 | 71 | 1'082 | -72 |
| 75 | Chelyabinsk oblast | 2'560 | 165 | 422 | 200 | 513 | 91 |
| 1 | Altai Krai | 3'682 | 148 | 545 | 189 | 697 | 152 |
| 2 | Altai Republic | 4'004 | 191 | 765 | 204 | 817 | 52 |
| 4 | Krasnoyarsk Krai | 71'378 | 131 | 9'346 | 170 | 12'135 | 2'789 |
| 25 | Irkutsk oblast | 43'838 | 167 | 7'305 | 193 | 8'467 | 1'162 |
| 32 | Kemerovo oblast | 5'657 | 133 | 750 | 248 | 1'402 | 652 |
| 50 | Novosibirsk oblast | 4'683 | 117 | 548 | 164 | 766 | 219 |
| 52 | Omsk oblast | 4'564 | 136 | 620 | 179 | 819 | 199 |
| 69 | Tomsk oblast | 19'324 | 148 | 2'859 | 218 | 4'204 | 1'345 |
| 93 | Tuva Republic | 3'108 | 145 | 452 | 184 | 573 | 121 |
| 95 | Khakassia Republic | 2'979 | 157 | 467 | 211 | 629 | 162 |
| 5 | Primorski Krai | 12'411 | 151 | 1'876 | 218 | 2'709 | 834 |
| 8 | Khabarovsk Krai | 31'833 | 114 | 3'630 | 146 | 4'650 | 1'020 |
| 10 | Amur oblast | 20'474 | 95 | 1'935 | 125 | 2'556 | 621 |
| 21 | Jewish a. oblast | 1'634 | 122 | 200 | 160 | 261 | 61 |
| 30 | Kamchatka oblast | 4'956 | 83 | 412 | 83 | 412 | 0 |
| 35 | Chukotka a.okr. | 1'781 | 31 | 56 | 41 | 73 | 17 |
| 44 | Magadan oblast | 7'495 | 44 | 327 | 42 | 317 | -10 |
| 64 | Sakhalin oblast | 5'608 | 114 | 637 | 191 | 1'071 | 434 |
| 76 | Zabaykalsky Krai | 23'772 | 101 | 2'408 | 138 | 3'272 | 864 |
| 81 | Buryatia Republic | 14'941 | 118 | 1'768 | 151 | 2'261 | 493 |
| 98 | Sakha Republic | 67'826 | 72 | 4'899 | 89 | 6'021 | 1'123 |
|  | Entire Russia | **575'689** | **123** | **70'578** | **164** | **94'211** | **23'633** |

Table S4. Example of calibrating data (attached to the article)

| Admin ID | Plot ID | GSV_GT | GSV_GB | GSV_CCI | PFT ID | Zone ID |
| --- | --- | --- | --- | --- | --- | --- |
| 47 | 77602003 | 14 | 38 | 75 | 5 | 6 |
| 47 | 77602006 | 216 | 247 | 281 | 4 | 7 |
|  | … |  |  |  |  |  |

Admin ID – unique ID of administrative region (see names in the Table S2);

Plot ID – unique plot ID;

GSV_GT – GSV estimation at NFI or FOS ground plot;

GSV_GB – GlobBiomass GSV, m³ ha^-1^;

GSV_CCI – CCI BIOMASS GSV, m³ ha^-1^;

PFT ID – forest type from Copernicus land cover map: 1 – needle-leaved evergreen, 3 – needle-leaved-deciduous (Larch), 4 – broad-leaved deciduous, 5 – mixed forest;

Zone ID – Bioclimatic zone: 2 – Forest-tundra, 3 – northern taiga, 4 – middle taiga, 5 – south taiga, 6 – temperate forest, 7 – forest-steppe.

Table S5. Predictive performance of the candidate models

| Models | Fit | | Cross-Validation | | |
| --- | --- | --- | --- | --- | --- |
|  | MAE | MSE | ME | MAE | MSE |
| Linear | 57.3 | 6813.9 | -0.0006 | 57.3 | 6821.2 |
| Linear, corrected | 56.8 | 6796.5 | 6.1659 | 58.6 | 6831.8 |
| Log-linear | 92.7 | 330161.9 | 122.9485 | 157.5 | 875828.5 |
| Log-Log | 60.5 | 8731.1 | 37.5733 | 72.1 | 8908.3 |
| Quadratic | 55.8 | 6657.1 | 0.0143 | 55.8 | 6677.4 |
| Quadratic, corrected | 60.1 | 21600.3 | 61.56289 | 93.2 | 588839.2 |
| Random Forest | 63.0 | 8515.8 | 67.2455 | 100.0 | 18623.4 |

Table S6. Coefficients of the linear model

|  | Mean | S.E. | 95% CI | |
| --- | --- | --- | --- | --- |
| $\boldsymbol{a}_{\mathbf{0},\mathbf{2}}$ | 39.0710 | 8.8204 | 22.096 | 56.9697 |
| $\boldsymbol{a}_{\mathbf{0},\mathbf{3}}$ | 37.8063 | 7.3266 | 23.8254 | 53.0858 |
| $\boldsymbol{a}_{\mathbf{0},\mathbf{4}}$ | 54.0162 | 7.3043 | 39.8089 | 69.2167 |
| $\boldsymbol{a}_{\mathbf{0},\mathbf{5}}$ | 80.9715 | 10.4694 | 61.0445 | 101.7536 |
| $\boldsymbol{a}_{\mathbf{0},\mathbf{6}}$ | -5.7500 | 17.059 | -40.1189 | 27.4017 |
| $\boldsymbol{a}_{\mathbf{0},\mathbf{7}}$ | 45.2302 | 21.8628 | 4.3295 | 89.8728 |
| $\boldsymbol{b}_{\mathbf{0},\mathbf{3}}$ | -41.0807 | 8.2914 | -58.0677 | -24.6549 |
| $\boldsymbol{b}_{\mathbf{0},\mathbf{4}}$ | -19.5794 | 9.8565 | -38.7136 | -0.0038 |
| $\boldsymbol{b}_{\mathbf{0},\mathbf{5}}$ | -39.6959 | 8.0703 | -56.2026 | -24.2198 |
| $\boldsymbol{a}_{\mathbf{1},\mathbf{2}}$ | 0.2009 | 0.2152 | -0.2361 | 0.6309 |
| $\boldsymbol{a}_{\mathbf{1},\mathbf{3}}$ | 0.3561 | 0.2076 | -0.0628 | 0.7533 |
| $\boldsymbol{a}_{\mathbf{1},\mathbf{4}}$ | 0.5647 | 0.2075 | 0.1704 | 0.9721 |
| $\boldsymbol{a}_{\mathbf{1},\mathbf{5}}$ | 0.2443 | 0.2193 | -0.1895 | 0.6886 |
| $\boldsymbol{a}_{\mathbf{1},\mathbf{6}}$ | 0.2660 | 0.2528 | -0.2260 | 0.7445 |
| $\boldsymbol{a}_{\mathbf{1},\mathbf{7}}$ | 0.6600 | 0.3872 | -0.0772 | 1.4485 |
| $\boldsymbol{b}_{\mathbf{1},\mathbf{3}}$ | 0.5866 | 0.1158 | 0.3735 | 0.8227 |
| $\boldsymbol{b}_{\mathbf{1},\mathbf{4}}$ | 0.3758 | 0.1218 | 0.1322 | 0.6107 |
| $\boldsymbol{b}_{\mathbf{1},\mathbf{5}}$ | 0.4410 | 0.1223 | 0.2005 | 0.6768 |
| $\boldsymbol{a}_{\mathbf{2},\mathbf{2}}$ | -0.2980 | 0.2479 | -0.7908 | 0.1745 |
| $\boldsymbol{a}_{\mathbf{2},\mathbf{3}}$ | -0.1600 | 0.2460 | -0.6208 | 0.3486 |
| $\boldsymbol{a}_{\mathbf{2},\mathbf{4}}$ | -0.5910 | 0.2439 | -1.0622 | -0.0984 |
| $\boldsymbol{a}_{\mathbf{2},\mathbf{5}}$ | -0.9912 | 0.2568 | -1.4774 | -0.4804 |
| $\boldsymbol{a}_{\mathbf{2},\mathbf{6}}$ | 0.2465 | 0.2647 | -0.2428 | 0.7503 |
| $\boldsymbol{a}_{\mathbf{2},\mathbf{7}}$ | -0.0707 | 0.3992 | -0.8172 | 0.7266 |
| $\boldsymbol{b}_{\mathbf{2},\mathbf{3}}$ | 0.7313 | 0.0949 | 0.5462 | 0.9190 |
| $\boldsymbol{b}_{\mathbf{2},\mathbf{4}}$ | 0.4373 | 0.0937 | 0.2576 | 0.6262 |
| $\boldsymbol{b}_{\mathbf{2},\mathbf{5}}$ | 0.3013 | 0.0916 | 0.1286 | 0.4833 |
| $\boldsymbol{a}_{\mathbf{3},\mathbf{2}}$ | 0.0049 | 0.0022 | 0.0007 | 0.0097 |
| $\boldsymbol{a}_{\mathbf{3},\mathbf{3}}$ | -0.0012 | 0.0021 | -0.0056 | 0.0030 |
| $\boldsymbol{a}_{\mathbf{3},\mathbf{4}}$ | -0.0007 | 0.0021 | -0.0053 | 0.0033 |
| $\boldsymbol{a}_{\mathbf{3},\mathbf{5}}$ | 0.0016 | 0.0021 | -0.0029 | 0.0058 |
| $\boldsymbol{a}_{\mathbf{3},\mathbf{6}}$ | -0.0024 | 0.0022 | -0.0070 | 0.0018 |
| $\boldsymbol{a}_{\mathbf{3},\mathbf{7}}$ | -0.0025 | 0.0027 | -0.0080 | 0.0027 |
| $\boldsymbol{b}_{\mathbf{3},\mathbf{3}}$ | -0.0050 | 0.0005 | -0.0060 | -0.0039 |
| $\boldsymbol{b}_{\mathbf{3},\mathbf{4}}$ | -0.0026 | 0.0005 | -0.0037 | -0.0016 |
| $\boldsymbol{b}_{\mathbf{3},\mathbf{5}}$ | -0.0021 | 0.0005 | -0.0031 | -0.0011 |


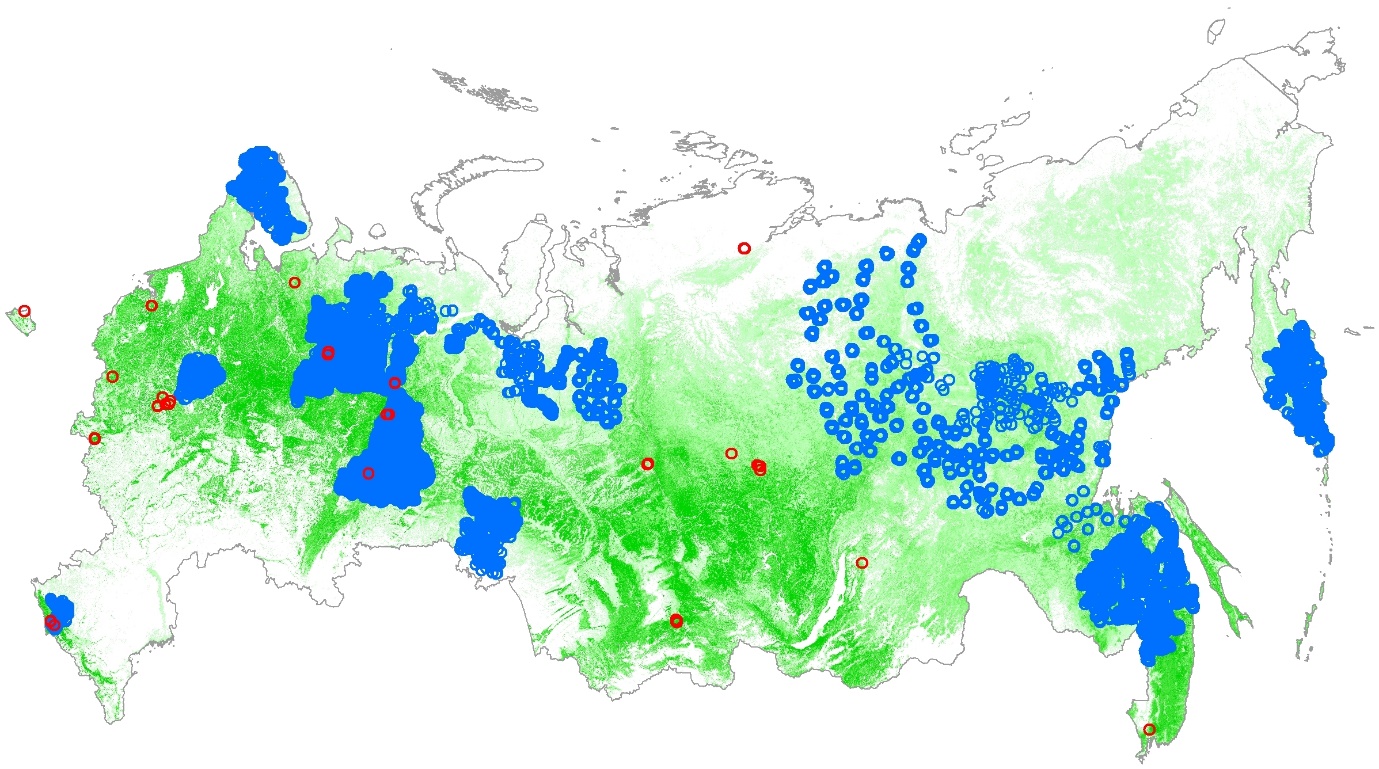


Figure S1. Distribution of growing stock volume represented by intensity of green color (Santoro et al., 2018), NFI plots (blue dots) and FOS plots (red dots) (Generated by Esri ArcGIS Desktop v.10.7, URL: https://desktop.arcgis.com/en/arcmap/)

Figure S2. Histogram of GSV from the GlobBiomass, CCI Biomass project, the dataset of ground sample plots and the State Forest Register (SFR)

| 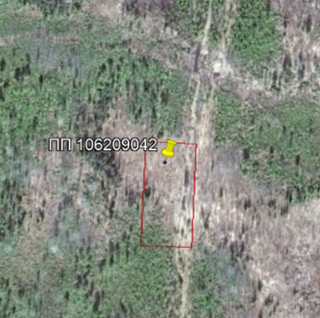  a) | 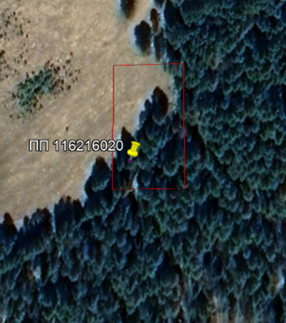  b) |
| --- | --- |

Figure S3. Example of data screening (Screenshots of DigitalGlobe imagery 2010 in Google Earth Pro v.7.3.3.7786, URL: https://www.google.com/earth/versions/#earth-pro):
a) NFI estimation – 1058 m^3^ hа^-1^, GB – 68 m^3^ ha^-1^; b) NFI – 522 m^3^ ha^-1^, GB – 41 m^3^ ha^-1^ (neighbor forest pixels – 250 m^3^ ha^-1^). Red rectangle – GB pixel, approximately 100х50 m.


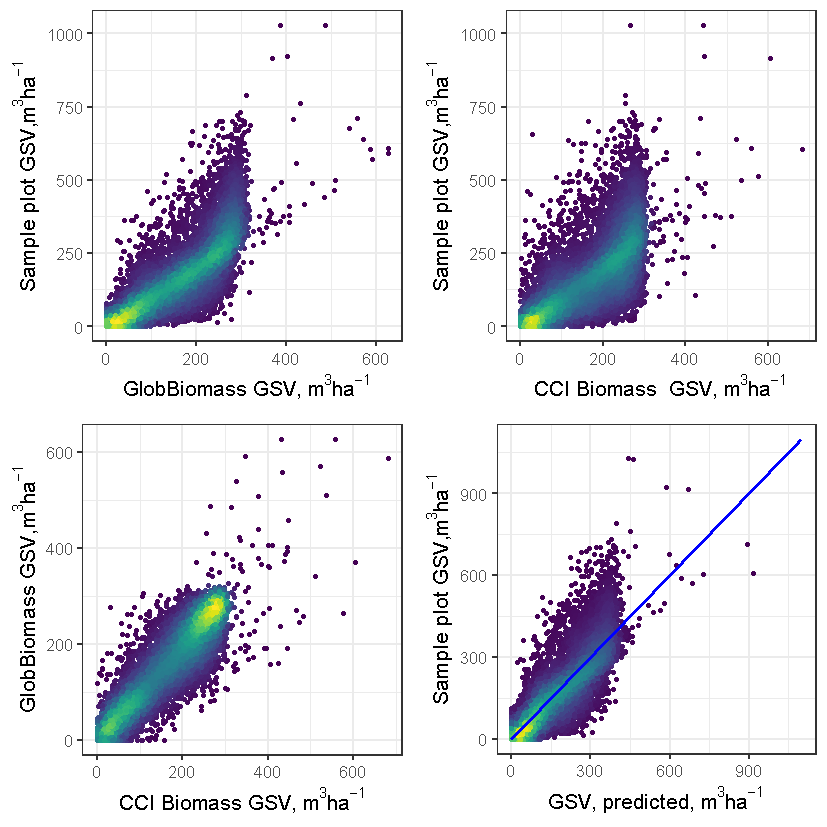


d

c

b

a

Figure S4. Scatter plot (a) GlobBiomass GSV against ground GSV; (b) GlobBiomass GSV against CCI Biomass GSV; (c) Ground GSV against CCI Biomass GSV; (d) Ground GSV against predicted GSV


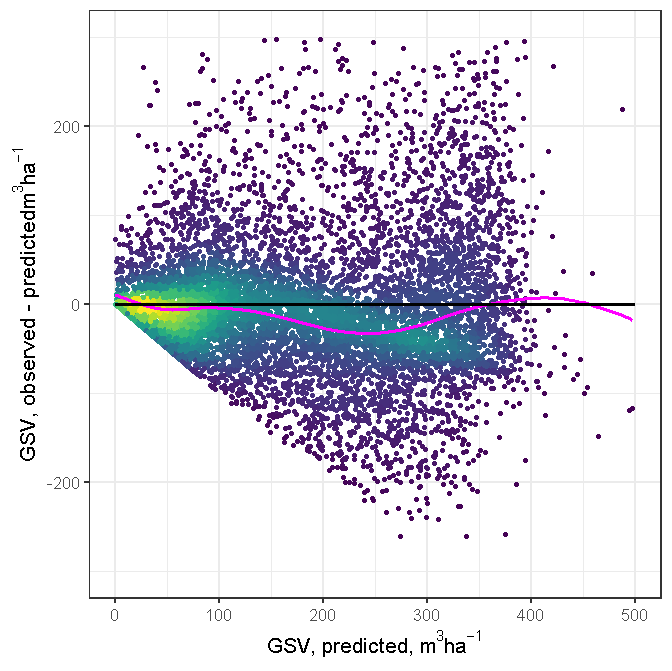


Figure S5. Linear model predictions vs. residuals for the calibrating data set

# S1. Selection of the regression model

The independent variables available for the prediction of GSV were GSV_GB_ (GlobBiomass), GSV_CCI_ (CCI Biomass), Zone and PFT (forest type). Because not all combinations of Zone and PFT were available in the calibrating data file, we were not able to consider a model allowing for the interaction of these two factors. We used a random forest algorithm to model the data, as well as fitting the following models outlined below:

Linear:

$$\boldsymbol{E(}\boldsymbol{GSV}_{\boldsymbol{GT}}\boldsymbol{)=}\left( \boldsymbol{a}_{\boldsymbol{0,zone}}\boldsymbol{+}\boldsymbol{b}_{\boldsymbol{0,PFT}} \right)\boldsymbol{+}\left( \boldsymbol{a}_{\boldsymbol{1,zone}}\boldsymbol{+}\boldsymbol{b}_{\boldsymbol{1,PFT}} \right)\boldsymbol{*}\boldsymbol{GSV}_{\boldsymbol{GB}}\boldsymbol{+}\left( \boldsymbol{a}_{\boldsymbol{2,zone}}\boldsymbol{+}\boldsymbol{b}_{\boldsymbol{2,PFT}} \right)\boldsymbol{*}\boldsymbol{GSV}_{\boldsymbol{CCI}}\boldsymbol{+}\left( \boldsymbol{a}_{\boldsymbol{3,zone}}\boldsymbol{+}\boldsymbol{b}_{\boldsymbol{3,zone}} \right)\boldsymbol{*}\boldsymbol{GSV}_{\boldsymbol{GB}}\boldsymbol{*}\boldsymbol{GSV}_{\boldsymbol{CCI}}\boldsymbol{,}$$

Log-linear:

$$\boldsymbol{E}\left( \log\left( \boldsymbol{GSV}_{\boldsymbol{GT}} \right) \right)\boldsymbol{=}\left( \boldsymbol{a}_{\boldsymbol{0,zone}}\boldsymbol{+}\boldsymbol{b}_{\boldsymbol{0,PFT}} \right)\boldsymbol{+}\left( \boldsymbol{a}_{\boldsymbol{1,zone}}\boldsymbol{+}\boldsymbol{b}_{\boldsymbol{1,PFT}} \right)\boldsymbol{*}\boldsymbol{GSV}_{\boldsymbol{GB}}\boldsymbol{+}\left( \boldsymbol{a}_{\boldsymbol{2,zone}}\boldsymbol{+}\boldsymbol{b}_{\boldsymbol{2,PFT}} \right)\boldsymbol{*}\boldsymbol{GSV}_{\boldsymbol{CCI}}\boldsymbol{+}\left( \boldsymbol{a}_{\boldsymbol{3,zone}}\boldsymbol{+}\boldsymbol{b}_{\boldsymbol{3,PFT}} \right)\boldsymbol{*}\boldsymbol{GSV}_{\boldsymbol{GB}}\boldsymbol{*}\boldsymbol{GSV}_{\boldsymbol{CCI}}\boldsymbol{,}$$

Log-log linear:

$$\boldsymbol{E}\left( \log\left( \boldsymbol{GSV}_{\boldsymbol{GT}} \right) \right)\boldsymbol{=}\left( \boldsymbol{a}_{\boldsymbol{0,zone}}\boldsymbol{+}\boldsymbol{b}_{\boldsymbol{0,PFT}} \right)\boldsymbol{+}\left( \boldsymbol{a}_{\boldsymbol{1,zone}}\boldsymbol{+}\boldsymbol{b}_{\boldsymbol{1,PFT}} \right)\boldsymbol{*}\log\left( \boldsymbol{GSV}_{\boldsymbol{GB}} \right)\boldsymbol{+}\left( \boldsymbol{a}_{\boldsymbol{2,zone}}\boldsymbol{+}\boldsymbol{b}_{\boldsymbol{2,PFT}} \right)\boldsymbol{*}\log\boldsymbol{GSV}_{\boldsymbol{CCI}}$$

And quadratic:

$$\boldsymbol{E(}\boldsymbol{GSV}_{\boldsymbol{GT}}\boldsymbol{)=}\left( \boldsymbol{a}_{\boldsymbol{0,zone}}\boldsymbol{+}\boldsymbol{b}_{\boldsymbol{0,PFT}} \right)\boldsymbol{+}\left( \boldsymbol{a}_{\boldsymbol{1,zone}}\boldsymbol{+}\boldsymbol{b}_{\boldsymbol{1,PFT}} \right)\boldsymbol{*}\boldsymbol{GSV}_{\boldsymbol{GB}}\boldsymbol{+}\left( \boldsymbol{a}_{\boldsymbol{2,zone}}\boldsymbol{+}\boldsymbol{b}_{\boldsymbol{2,PFT}} \right)\boldsymbol{*GSV}_{\boldsymbol{GB}}^{\boldsymbol{2}}\boldsymbol{+}$$

$$\left( \boldsymbol{a}_{\boldsymbol{3,zone}}\boldsymbol{+}\boldsymbol{b}_{\boldsymbol{3,PFT}} \right)\boldsymbol{*AGB+}\left( \boldsymbol{a}_{\boldsymbol{4}\boldsymbol{,zone}}\boldsymbol{+}\boldsymbol{b}_{\boldsymbol{4}\boldsymbol{,PFT}} \right)\boldsymbol{*}{\boldsymbol{GSV}_{\boldsymbol{CCI}}}^{\boldsymbol{2}}\boldsymbol{+}$$

$$\left( \boldsymbol{a}_{\boldsymbol{5,zone}}\boldsymbol{+}\boldsymbol{b}_{\boldsymbol{5,PFT}} \right)\boldsymbol{*}\boldsymbol{GSV}_{\boldsymbol{GB}}\boldsymbol{*}\boldsymbol{GSV}_{\boldsymbol{CCI}}\boldsymbol{.}$$

Since the linear and quadratic models allow for negative predictions, we have also considered “corrected” models, where the negative predictions were set to zero. The performance of the models described above was assessed via 20-fold cross-validation. Mean error (ME), mean absolute error (MAE), and mean squared error (MSE) were calculated in each case and are shown in Table S5. Less complex nested sub-models had poorer performance (and hence are not shown). Based on the predictive performance, the two best models by far were the linear and the linear-corrected model.

Because the residuals of the resulting model displayed strong heteroscedasticity, the estimated standard errors for the regression parameters could not be used to produce the confidence intervals for predictions. We have, therefore, used 1000 bootstrapped estimates to obtain the 95% confidence intervals for the administrative area-specific GSV density per ha.

# S2. R-script fitting the model and cross-validation

##########################################################################

# 25/11/2020 #

# Producing predictions and 95% based on weighted linear regression model #

# The weights reflect the differences between the frequency #

# distributions of GSV_GB in the calibrating data and the administrative data #

# #

# line-specific predictions #

# #

# by Elena Moltchanova #

##########################################################################

rm(list=ls())

library(data.table)

BOOT <- 1000 # number of bootstrapping sims

### reading in the calibrating data

data.trn <- read.csv("calibrating_db.csv")

# recasting RFT as a factor variable

data.trn$PFT <- as.factor(data.trn$PFT)

data.trn$Zone <- as.factor(data.trn$Zone)

### Preparing arrays for estimates

ID.LIST <- ADM.LIST <- MEAN <- MEAN.LO <- MEAN.HI <-

PRED.LO <- PRED.HI <- TOT.AREA <- MEAN.SE <- PRED.SE <- NULL

### reading in the admin data

# name of the file:

for(fj in 1:9){

fx <- paste("P:\\H\\collabs\\Dmitry\\biomass2020Dec\\NFI_RUS\\RUS_map_f",fj,".csv",sep="")

data.admin <- fread(fx)

data.admin$PFT <- as.factor(data.admin$PFT)

data.admin$Zone <- as.factor(data.admin$ZONE)

# the list of administrative areas in that dataset.

adm.list <- unique(data.admin$Admin)

for(j in 1:length(adm.list)){print(paste("file =", fj, "; admin[" ,j, "]=",adm.list[j],sep=" "))

# selecting the data

dt.tmp <- data.admin[data.admin$Admin==adm.list[j],]

ADM.LIST <- c(ADM.LIST,rep(adm.list[j],dim(dt.tmp)[1]))

tot.area <- sum(dt.tmp$AREA_HA)

#TOT.AREA <- c(TOT.AREA, tot.area)

### weights

dt.tmp$cat <- cut(dt.tmp$GSV_GB,seq(-1,1500,10))

data.trn$cat <- cut(data.trn$GSV_GB,seq(-1,1500,10))

wgt <- tapply(dt.tmp$AREA_HA,dt.tmp$cat,sum)

# this returns NAs if there are no records. turn those into 0:

wgt[is.na(wgt)] <- 0

mean.est.sx <- obs.est.sx <-

mean.est.sx2 <- obs.est.sx2 <- rep(0,dim(dt.tmp)[1])

### bootstrapping the calibrating data:

for(boot in 1:BOOT){print(boot)

dt.trn <- data.trn[sample(1:dim(data.trn)[1],size=dim(data.trn)[1],replace=T),]

wgt.orig <- table(dt.trn$cat)

dt.trn$wgt <- wgt[as.numeric(dt.trn$cat)]/wgt.orig[as.numeric(dt.trn$cat)]

# fitting a weighted reg model

Mw <- lm(GSV_ground ~ GSV_GB*GSV_CCI*(PFT+Zone), data=dt.trn, weights = wgt)

pred.wgt <- predict(Mw,newdata=dt.tmp)

# producing observation

pred.wgt.obs <- pmax(rnorm(length(pred.wgt),pred.wgt, sd=summary(Mw)$sigma),0)

mean.est.sx <- mean.est.sx+pmax(0,pred.wgt)

mean.est.sx2 <- mean.est.sx2+pmax(0,pred.wgt)^2

obs.est.sx <- obs.est.sx+pred.wgt.obs

obs.est.sx2 <- obs.est.sx+pred.wgt.obs^2

} # end of bootstrap

MEAN <- c(MEAN, mean.est.sx/BOOT)

MEAN.SE <- c(MEAN.SE, sqrt(mean.est.sx2/BOOT-(mean.est.sx/BOOT)^2))

PRED <- c(PRED, obs.est.sx/BOOT)

PRED.SE <- c(PRED.SE, sqrt(obs.est.sx2/BOOT-(obs.est.sx/BOOT)^2))

} # end of adm.list j-loop

} # end of the file loop

dt.out <- data.frame(admin = ADM.LIST[order(ADM.LIST)],

area = TOT.AREA[order(ADM.LIST)],

mean = MEAN[order(ADM.LIST)],

mean.lo = MEAN.LO[order(ADM.LIST)],

mean.hi = MEAN.HI[order(ADM.LIST)],

pred.lo = PRED.LO[order(ADM.LIST)],

pred.hi = PRED.HI[order(ADM.LIST)],

mean.se = MEAN.SE[order(ADM.LIST)],

pred.se = PRED.SE[order(ADM.LIST)])

write.csv(dt.out, file=" predicted_means_weighted_out.csv",

quote=F,row.names=F)

plot(dt.out$admin,dt.out$mean,ylim=c(0,400),pch=16)

arrows(dt.out$admin,dt.out$pred.lo,dt.out$admin,dt.out$pred.hi,angle=90,code=3,length=.025)

arrows(dt.out$admin,dt.out$mean.lo,dt.out$admin,dt.out$mean.hi,angle=90,code=3,length=.025,col='red')
